# Osteodifferentiation of Human Preadipocytes Induced by Strontium Released from Hydrogels

**DOI:** 10.1155/2012/865291

**Published:** 2012-08-09

**Authors:** Valeria Nardone, Sergio Fabbri, Francesca Marini, Roberto Zonefrati, Gianna Galli, Annamaria Carossino, Annalisa Tanini, Maria Luisa Brandi

**Affiliations:** Department of Internal Medicine, University of Florence, 50139 Florence, Italy

## Abstract

In recent years, there has been an increasing interest in interactive application principles of biology and engineering for the development of valid biological systems for tissue regeneration, such as for the treatment of bone fractures or skeletal defects. The application of stem cells together with biomaterials releasing bioactive factors promotes the formation of bone tissue by inducing proliferation and/or cell differentiation. In this study, we used a clonal cell line from human adipose tissue-derived mesenchymal stem cells (hADSCs or preadipocytes), named PA2-E12, to evaluate the effects of strontium (Sr^2+^) released in the culture medium from an amidated carboxymethylcellulose (CMCA) hydrogel enriched with different Sr^2+^ concentrations on osteodifferentiation. The osteoinductive effect was evaluated through both the expression of alkaline phophatase (ALP) activity and the hydroxyapatite (HA) production during 42 days of induction. Present data have shown that Sr^2+^ released from CMCA promotes the osteodifferentiation induced by an osteogenic medium as shown by the increase of ALP activity at 7 and 14 days and of HA production at 14 days. In conclusion, the use of biomaterials able to release *in situ* osteoinductive agents, like Sr^2+^, could represent a new strategy for future applications in bone tissue engineering.

## 1. Introduction

The repair of large bone defects, due to trauma, tumors, and/or congenital malformations is a global health problem and a major challenge for orthopaedic surgery [1–4]. Current treatment options include surgical reconstruction by organ/tissue transplantation of autografts/allografts. These traditional methods are often associated with limited availability in autografts, and risk of immunogenicity, infection, and local pain [5, 6]. Today, tissue engineering by artificial tissue grafts represents a valid alternative for overcoming the therapeutic and methodological limitations of current therapy [7–11]. The aim of tissue engineering is to produce functional tissues *in vitro *[1], in order to improve *in vivo* regeneration using cells, biodegradable biomaterials/scaffolds, and bioactive factors [10, 12–16]. The advantage of this approach is that it can reduce the number of surgical operations and the time of recovery for solving the clinical problems.

Scaffolds are key components acting as a mold for interaction with the cells, also providing support for cell adhesion, growth, and differentiation. A good scaffold has to be osteoconductive (to induce the cells to adhere, migrate, and proliferate), osteoinductive (to be able to induce proliferation of undifferentiated cells and their subsequent differentiation into osteoblasts), biocompatible, and biodegradable [17]. Moreover, a scaffold must possess mechanical properties similar to the native tissue to be repaired. A further requirement for a scaffold, particularly in bone engineering, is a controllable interconnected porosity to promote engraftment, proliferation, and migration of bone cells, as well as synthesis of the extracellular matrix (ECM), vascularization of the ingrown tissue, and interconnection between the implant and the bone tissue, in order to ensure mechanical stability [14, 18, 19]. In addition, the mechanical properties of the scaffold must be sufficient and not collapse during handling and during the patient's normal activities. Finally, the scaffold must be easily sterilizable to prevent infections [20].

The other component for the engineering of a viable tissue construct is the use of cell therapy. Mesenchymal stem cells (MSCs), given their osteoblast-driven differentiation potential, represent the most suitable cell source in bone regeneration therapies. MSCs are multipotent cells identified in numerous tissues such as bone marrow, fat, placenta, umbilical cord, human amniotic fluid, dental pulp, and skeletal muscle [21–28]. Many studies have demonstrated the usefulness of MSCs for regenerative medicine, in particular in osteoarticular disorders [29]. MSCs, isolated from adult bone marrow (BMMSCs), can be induced *in vitro* and *in vivo* to differentiate into various mesenchymal lineages (bone, cartilage, tendon, adipose tissue, and muscle). These cells can also differentiate into nonmesenchymal cell lines, such as endothelial cells [30], cardiac myoblasts [31], neuronal cells [32], and hepatocytes [33]. BMMSCs have been demonstrated to stimulate bone formation in skeletal defects and nonunion, through cytokines and growth factors secreted by the transplanted cells [34, 35]. Recent studies have demonstrated that also human adipose tissue-derived mesenchymal stem cells (hADSCs) are able to differentiate into active osteoblasts, like their bone marrow counterpart (hBMMSCs) [36–38]. These characteristics, together with the greater quantity obtainable and the low invasiveness of fat sampling procedure, make adipose tissue an excellent cell source for bone regeneration [38].

Several studies have investigated the applicability of hydrogels, water-soluble polymers which swell to form a gel-like substance upon exposure to water [39, 40], acting as biodegradable and biocompatible scaffolds for bone grafts and cartilage regeneration [41–48]. *In vitro* studies have demonstrated that amidated carboxymethyl cellulose (CMCA) hydrogel is a potential filler for cartilage defects. Normal human articular chondrocytes seeded on CMCA [48] promote synthesis of ECM components, significantly increasing production of both type II collagen and aggregan, the hallmark proteoglycan for hyaline cartilage [49]. These data suggest that CMCA hydrogels could represent a good support for tissue engineering in osteoarticular disorders [48]. Currently, attention has been focused on the use of scaffolds enriched with bioactive factors such as biologically active proteins, growth factors, hormones, cytokines, and drugs capable of inducing cell proliferation and/or differentiation [50–54]. Moreover, the addition of anti-inflammatory drugs and antibiotics can make the prevention of infections after surgery possible [55].

An agent registered as an antifracture drug is strontium ranelate (SR), whose active component on bone remodelling is the Sr^2+^ ion [56–58]. Unlike all the other treatments for osteoporosis, SR has a dual effect on bone remodelling, being able, simultaneously, to stimulate osteoblast-mediated bone formation and to inhibit osteoclast-induced bone resorption [59]. Indeed, SR stimulates *in vitro* osteoblastic differentiation markers such as alkaline phosphatase (ALP), bone sialoprotein, and osteocalcin (OCN) and also inhibits the proliferation of osteoclast precursors as well as osteoclastogenesis [60, 61].

*In vitro* studies have shown that the use of biomaterials enriched with Sr^2+^ is promising. In fact, strontium-doped-calcium-polyphosphate-(SCPP) based bioceramic scaffolds combined with the rat osteosarcoma cell line ROS17/2,8 promoted cell proliferation and induced mRNA expression and release of two angiogenic factors, vascular endothelial growth factor (VEGF) and basic fibroblast growth factor (bFGF) [62–64].

The encouraging results obtained by the use of cells combined with hydrogels and by the use of scaffolds enriched with Sr^2+^ on the *in vitro* osteogenic differentiation of hADSCs, prompted us to perform a study on the combined use of hydrogels and Sr^2+^, to assess its potential for future applications in bone tissue engineering.

## 2. Materials and Methods

### 2.1. CMCA Hydrogel and CMCA Hydrogel Enriched with Sr^2+^ Preparation

The CMCA hydrogel and CMCA hydrogel enriched with Sr^2+^ used in this study were obtained from BioSuMa (Lima Corporate S.p.a., Villanova di San Daniele del Friuli, Italy). The procedure for the realization of amidic derivative of CMC-based hydrogel (CMCA) was previously reported [65]. The kinetics of degradation *in vitro* of CMCA hydrogel were evaluated at 7 days by hyialuronidase and *β*-mannosidase enzymes with percentages of degradation, respectively, of 7% and 32% [experimental data not shown, provided by R. Barbucci (C.R.I.S.M.A) University of Siena]. The preparation of CMCA enriched with Sr^2+^ was carried out by bulge of gel in aqueous solutions of 3, 30, 300 *μ*M and 3 mM SrCl_2_, exploiting the property of hydrogel to incorporate a large quantity of water. The hydrogel enriched with Sr^2+^ was stratified in transwell 6-well Millicell inserts (Millipore) with 2.5 cm diameter, dehydrated, and sterilized by ethylene oxide. The evaluation of the amount of Sr^2+^ incorporated in the hydrogel and then released into the medium was carried out by inductively coupled plasma mass spectrometry (ICPMS) technique that uses an ICP torch to produce the ionization, and a mass spectrometer to separate and detect the ions produced [66].

### 2.2. Cell Culture

A primary cell line of hADSCs, named PA2, previously cultured and characterized for its multi-potency in our laboratory [39], was plated on tissue culture polystyrene (tPS) substrate at 37°C in humidified atmosphere with 5% CO_2_ in growth medium (GM) [Ham's F12 Coon's modification medium supplemented with 10% fetal calf serum (FCS), 100 IU/mL penicillin, 100 *μ*g/mL streptomycin, and 1 ng/mL basic fibroblast growth factor (bFGF)]. The medium was refreshed twice a week and cells were used for further subculturing or cryopreservation upon reaching 5 × 10^3^ cells/cm^2^.

### 2.3. Cell Cloning

The primary cell line PA2 at the 3rd passage were used for cell cloning. Cells in active phase of growth were cloned by the dilution plating technique. Cells were detached with trypsin 1 : 250 0.4 mg/mL in Dulbecco's phosphate-buffered saline (DPBS) without Ca^2+^ without Mg^2+^ with EDTA 0.2 mg/mL and with glucose 1 mg/mL, resuspended in Coon's medium + 20% FCS. The cell suspension was diluted to a concentration of 10 cells/mL in the following cloning medium: Coon's medium + 20% FCS supplemented with 25% conditioned medium prepared from human fetal fibroblast culture. The cell suspension was maintained in agitation and 0.1 mL was rapidly distributed per well of a 96-multiwell plate. Each well was carefully observed and the wells containing only one cell were scored. The cloning culture was incubated at 37°C in humidified air with 5% CO_2_. When colonies reached the consistency of 500–600 cells, they were detached, collected, and first transferred in 24-multiwell plates and subsequently expanded in 60 mm and 100 mm dishes. Seven finite clonal lines, named PA2-C5, PA2-D4, PA2-E12, PA2-F2, and PA2-H8, were obtained from the PA2 cell line. PA2-E12 was chosen among these finite clonal cell lines for its high proliferative capacity.

### 2.4. Clonal Cell Line Characterization

The characterization of PA2-E12 finite clonal cell line, to verify its multi-potency, was performed by studying the adipogenic and osteogenic differentiation, as previously described [38].

### 2.5. Adhesion and Morphology Analysis

CMCA hydrogel was sterilized by ethanol, balanced in GM, and afterwards distributed on tPS. The plates coated with CMCA hydrogel were preincubated at 37°C in humidified atmosphere with 5% CO_2_ in GM for 4 h. Thereafter, PA2-E12 cells were cultured in GM and seeded on CMCA hydrogel. PA2-E12 cells cultured on CMCA hydrogel were evaluated for cell morphology and capacity to adhere to CMCA hydrogel after 1, 3, 6, 9, 12, and 15 days from seeding. Total cellular RNA was isolated both from cells cultured in GM on tPS and cells cultured in GM on CMCA hydrogel during time of culture.

### 2.6. Osteogenic Differentiation in Presence of CMCA Hydrogel Enriched with Sr^2+^

PA2-E12 cells were previously seeded at semiconfluence on tPS in 6-multiwell plates at a cell density of 1 × 10^4^ cells/cm^2^ in GM. After 3 days at achievement of confluence, the cells were differentiated by on osteogenic medium (OM): Coon's medium supplemented with 10% FCS, 100 IU/mL penicillin, 100 *μ*g/mL streptomycin, 10 nM dexamethasone, 10 mM *β*-glycerophosphate, and 200 mM sodium L-ascorbyl-2-phosphate. The osteogenic differentiation was carried out in OM in the presence of 3 *μ*M–3 mM Sr^2+^. In parallel, experiments in OM were carried out in the presence of transwell containing CMCA hydrogel enriched with 0, 3, 30, 300 *μ*M and 3 mM Sr^2+^. The medium was refreshed twice a week. The expression of the osteoblastic phenotype was evaluated quantitatively by ALP activity and by hydroxyapatite (HA) production at different times from 1 to 42 days of culture and respective values were normalized by DNA content/well. For ALP assay, each well was incubated with 500 *μ*L of 4-methylumbelliferyl phosphate in 280 mM Tris-HCl buffer pH 9.0 for 15 min at 37°C. The reaction was stopped by the addition of 2 mL of 0.1 M NaOH. ALP activity was measured with a spectrofluorometer LS55 (PerkinElmer) at 365 nm *λ* excitation and 445 nm *λ* emission and expressed in *μ*U ALP/ng DNA using a standard curve of 4-methylumbelliferone 50 nM–10 *μ*M in 280 mM Tris-HCl buffer pH 9.0. For HA assay, cells were grown in OM containing 1 mg/mL calcein, fixed and washed. Afterwards, each well was incubated with 2 mL of 50 mM NaEDTA for 30 min at 37°C, then the fluorescence was measured with spectrofluorometer LS55 (PerkinElmer) at 494 nm *λ* excitation and 517 nm *λ* emission and expressed in *μ*g HA/ng DNA using a standard curve of HA 25 ng/mL–500 *μ*g/mL solubilized in 50 mM NaEDTA. A cytochemical evaluation for ALP was carried out at 14 days of culture using a method of simultaneous coupling between naphthol and diazonium salt to obtain an azoic dye. The cells were washed with DPBS (two times), stained with a specific dye mixture (5 mg Naphthol-AS-MX phosphate sodium salt dissolved in 1 mL dimethyl sulfoxide), 40 mg fast red violet LB dissolved in 49 mL Tris-HCl buffer 280 mM pH 9.0 for 30 min at 37°C. Then, the cells were washed with DPBS (two times, fixed in 4% paraformaldehyde (PFA)/DPBS for 15 min and washed with ultrapure water three times). ALP+ cells were stained in red. No staining was carried out to highlight HA deposits that result in black because of their optical density property in transmitted light.

### 2.7. Statistical Analysis

The statistical significance of differences between mean values of ALP activity and of HA deposits production between controls and stimuli were evaluated by two-tailed Student's *t*-test on experiments repeated three times and carried out in quadruplicate.

## 3. Results

### 3.1. Adhesion and Cellular Morphology

**Figure 1:**
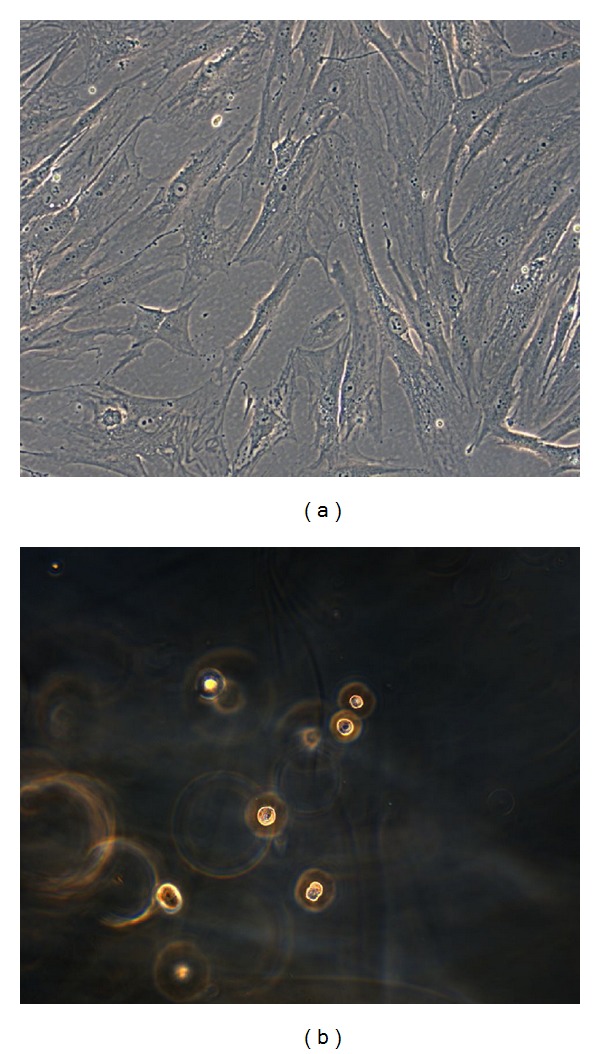
(a) Phase-contrast microscopy (20x objective) of PA2-E12 cultured in GM on tPS after 1 day of culture. (b) Phase-contrast microscopy (20x objective) of PA2-E12 on CMCA hydrogels after 1 day of culture.

PA2-E12 cultured in GM on tPS (controls) showed an optimal adhesion to the surface with a fusiform shape after 1 day of culture (Figure 1(a)). PA2-E12 cultured on CMCA hydrogels showed a round morphology and a limited adhesion on CMCA hydrogels after 1 day of culture (Figure 1(b)). Total RNA of cells cultured in GM on tPS increased during time of culture (1–15 days) with cellular proliferation and the cells maintained a fusiform shape. Total RNA of cells adherent to hydrogel was constant during time of culture (1–15 days), but it was near to the lower limit detectable due to low number of cells adherent to hydrogel.

### 3.2. Sr^2+^ Loading on CMCA Hydrogel and Sr^2+^ Release and Accumulation in the Culture Medium

The percentage of Sr^2+^ incorporation in the CMCA hydrogel was 95% of the total exposure and the Sr^2+^ released in the culture medium at 37°C for each hour resulted to be of the order of 2% the ion incorporated into the hydrogel. The Sr^2+^ was accumulated in the culture medium until the replacement of the old medium with fresh medium (Table 1). During time of culture of cells, CMCA hydrogel showed no alteration or degradation signs in the structure 3D by microscopic observation. 

### 3.3. Effects of Sr^2+^ Dissolved Directly in OM and of Sr^2+^ Released from CMCA Hydrogel in OM on ALP Activity

**Figure 2:**
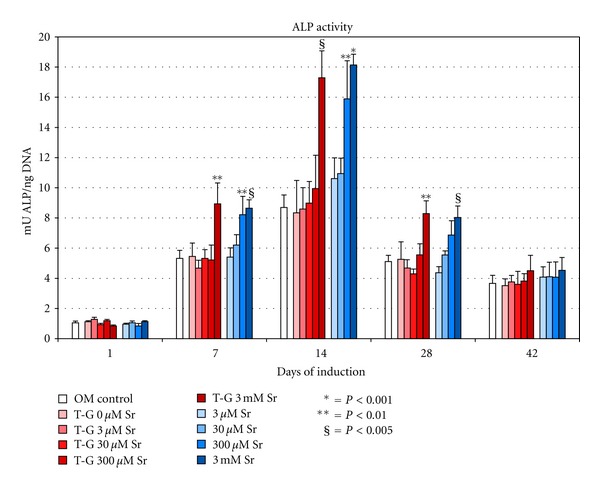
Quantitative analysis of ALP enzymic activity in PA2-E12 cultured on tPS from 1 to 42 days in OM in the presence of transwell containing CMCA hydrogel (T-G) enriched with scalar concentrations of strontium (T-G 0, 3, 30, 300 *μ*M and 3 mM Sr^2+^) or in OM only containing scalar concentrations of strontium (3, 30, 300 *μ*M and 3 mM Sr^2+^). The control is represented by cells cultured in OM without Sr^2+^.

An increasing ALP activity was measured from 1 to 14 days in all samples with a decreasing trend afterwards. The production of ALP was always present in all samples after 42 days with mean increases of 282% compared to 1 day of induction. Significant increases of ALP activity versus controls were observed in cells cultured in OM in presence of Sr^2+^ released from CMCA hydrogel enriched with 3 mM Sr^2+^ (corresponding to culture medium concentrations of 600 *μ*M after 7 days of culture, 20 *μ*M after 14 days, and 20 nM after 28 days) with maximum percent increase of 99% after 14 days (Figure 2). Conversely, in CMCA hydrogel enriched with 0, 3, 30 and 300 *μ*M Sr^2+^, no effect was observed on ALP induction (Figure 2).

Similarly, in cells cultured in OM containing 300 *μ*M and 3 mM Sr^2+^, significant increases compared to controls were observed after 7 and 14 days (maximal increases after 14 days for 300 *μ*M and 3 mM Sr^2+^, resp., 85% and 106%), with a prolonged effect up to 28 days only in the presence of 3 mM Sr^2+^. As expected, significant increases of ALP activity were not observed in cells cultured in OM containing 3 or 30 *μ*M Sr^2+^. After 42 days of induction, ALP activity decreased in all samples, without significant differences compared to controls (Figure 2).

### 3.4. Effects of Sr^2+^ Dissolved Directly in OM and of Sr^2+^ Released from CMCA Hydrogel in OM on the Formation HA Deposits

**Figure 3:**
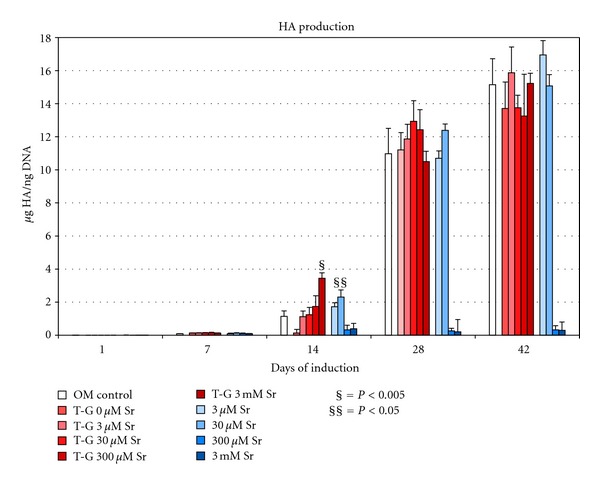
Quantitative analysis of the formation of HA deposits in PA2-E12 cultured on tPS from 1 to 42 days in OM in presence of transwell containing CMCA hydrogel (T-G) enriched with scalar concentrations of strontium (T-G 0, 3, 30, 300 *μ*M and 3 mM Sr^2+^) or in OM only containing scalar concentrations of strontium (3, 30, 300 *μ*M and 3 mM Sr^2+^). The control is represented by cells cultured in OM without Sr^2+^.

The formation of HA deposits begins to be observed after 14 days, increasing during time up to 42 days of culture. Low concentrations of Sr^2^ directly dissolved in OM did not modify the progression of HA deposition, while at 300 *μ*M and 3 mM Sr^+2^ the accumulation of HA was dramatically reduced at all analyzed times. Conversely, for cells cultured with 30 *μ*M Sr^2+^ directly dissolved in OM, significant increases of HA deposits compared to control were observed only after 14 days with percent increase of +98%. Similarly, at 14 days, significant increases of HA deposits formation were observed for cells cultured in OM in the presence of Sr^2+^ released from the CMCA hydrogel enriched with 3 mM Sr^+2^ and accumulated in the culture medium at a final concentration of 20 *μ*M Sr^+2^ (Table 1) with percent increase: 169% versus control. After 28 and 42 days, no significant differences compared to control were observed for cells cultured with Sr^2+^-enriched CMCA hydrogel (Figure 3).

### 3.5. ALP and HA Activity

**Figure 4:**
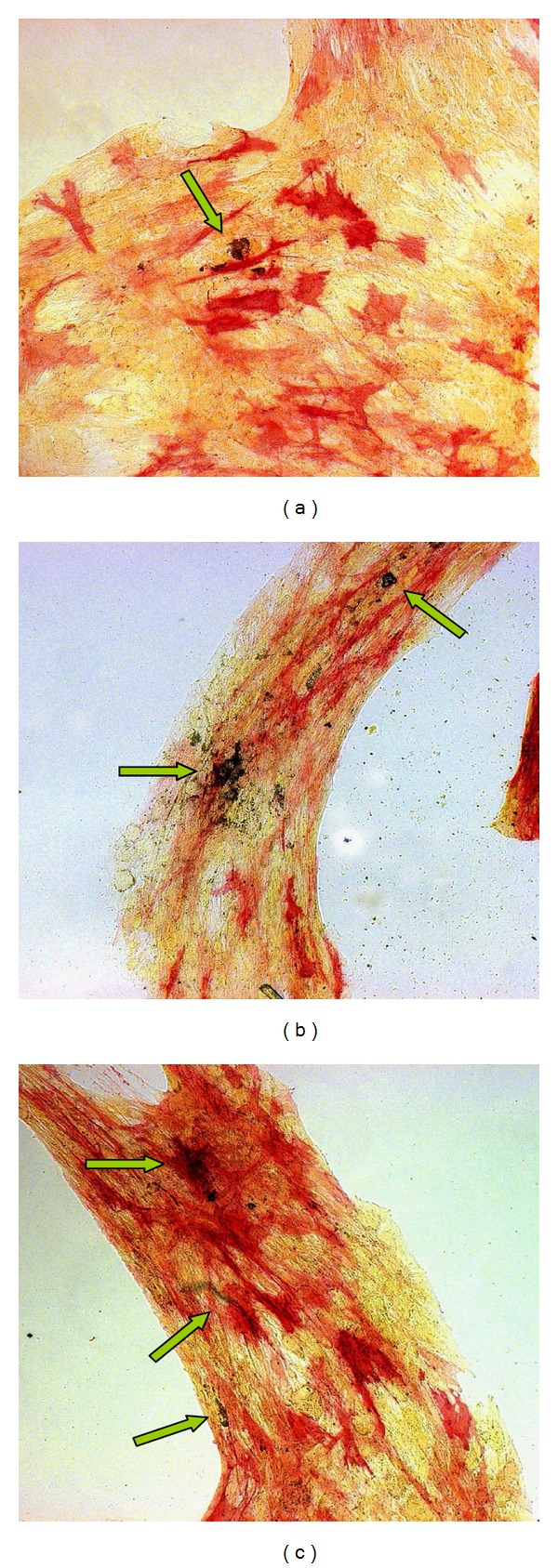
PA2-E12 cultured on tPS for 14 days in OM alone (a), in OM added with 30 *μ*M Sr^2+^ (b), and without in the presence of gel-enriched with 3 mM Sr^2+^ (c). Cells fixed and stained for ALP. Observation in brightfield microscopy (10x objective): ALP+ cells intensity in red, ALP− cells in yellow, and HA deposits in black (green arrows).

According to the results obtained with the quantitative ALP and HA analysis, 3 mM Sr^2+^ concentration loaded on CMCA hydrogel and 30 *μ*M Sr^2+^ concentration directly added to OM were used for light microscopy observation. A similar qualitative osteogenic differentiation was observed at 14 days in cells cultured on tPS in OM in the presence of 3 mM Sr^2+^-enriched CMCA hydrogel, in the presence of 30 *μ*M Sr^2+^ added directly to the OM, and in the presence of control OM alone (Figure 4). In all the three conditions an initial formation of HA deposits was observed with a higher density in the presence of Sr^2+^ ion, with cell groups at different staining intensity for ALP activity being present in all conditions, but more abundant in the presence of the Sr^2+^ ion(Figure 4).

## 4. Discussion

The interaction between stem cells and biomaterials represents an innovation in the tissue-engineered field for the replacement of damaged bone tissues, representing a great challenge for orthopaedic surgeons in the repair of bone and/or cartilage large defects. To offer the best opportunities for bone tissue repair innovation, the development of both novel biomaterials and ideal cell models is needed.

The design of biomimetic materials for the development of biomaterials is an area of great interest for tissue engineering applications [67–69]. Biomaterials can be coated with bioactive molecules that can serve as an artificial extracellular matrix (ECM) providing suitable background to promote cell adhesion and proliferation [70]. The surface modification of biomaterials, coated with bioactive molecules, can be made using long chains of ECM proteins such as fibronectin (FN) and laminin (LN), or a short peptide such as Arg-Gly-Asp (RGD) derived from FN and LN [53]. Hydrogels are able to protect drugs, peptides, and especially proteins against the potentially harsh surrounding microenvironment [71].

In this paper, CMCA hydrogels used in combination with PA2-E12, a clonal cell line obtained from a hADSCs line, did not result to be suitable to promote cell adhesion, as a consequence of basal chemical structure hydrogels, and determined a rounded cell morphology. In literature, data have shown that the hydrogel's surface must be chemically or biologically engineered (e.g., with the addition of adhesion ligands, short fragments of bioactive molecules) to obtain good bioactivity [72, 73].

The presence of CMCA hydrogel in the transwell for the PA2-E12 cells cultured on tPS did not modify the osteogenic characteristics of these cells, with no interference to the osteogenic differentiation process and without toxic effects. These data obtained on the biocompatibility have shown that CMCA hydrogels had the characteristics to be modified with the addition of bioactive molecules. In fact, we have modified the characteristics of hydrogel with addition of different concentrations of Sr^2+^ to investigate the effects of strontium release from CMCA hydrogel on the osteodifferentiation of PA2-E12 cells. The 3 mM Sr^2+^ concentration loaded on CMCA hydrogel was able to promote the osteodifferentiation of PA2-E12 cells, as shown by increased ALP activity at 7 and 14 days compared to OM. Moreover, at 7 days the response of ALP production in CMCA enriched with 3 mM Sr^2+^ was analogous to that of the cells cultured in OM with 3 mM Sr^2+^, probably due to an initial high release of Sr^2+^ in culture medium, able to more quickly direct the osteodifferentiation of the PA2-E12. CMCA hydrogel enriched with 3 mM Sr^2+^ was also able to promote the formation of HA deposits, as shown by increased HA activity at 14 days compared to OM. This response was similar to that obtained with the cells cultured in OM with 30 *μ*M Sr^2+^, probably due to the similar Sr^2+^ concentrations present in the culture medium. In fact, at 14 days the Sr^2+^ concentrations accumulated in the culture medium after release from the hydrogel enriched with 3 mM of Sr^2+^ was about 20 *μ*M. These results are confirmed by qualitative analysis of ALP activity and of HA formation for PA2-E12 cells with light microscopy observation after staining. In fact, 3 mM Sr^2+^ loaded in CMCA and 30 *μ*M Sr^2+^ directly added to OM, the more responsive concentrations of Sr^2+^ resulting from the quantitative analysis, were able to increase the number of ALP+ cells and the density of HA deposits compared to control at 14 days of osteoinduction.

The higher doses (e.g., 300 *μ*M and 3 mM) of Sr^2+^ in the cells cultured on tPS seem to inhibit the formation of HA deposits presupposing alterations of the physicochemical properties in the structure of hydroxyapatite crystal up to impede its formation [74].

In conclusion, multiple physical, chemical, and biological mechanisms are involved in tissue regeneration *in vitro* and *in vivo* using biomaterials [75]. The three-dimensionality, the biocompatibility, and biodegradability of hydrogel and its chemical surface characteristics, due to addition of biofactors able to promote fundamental cell biological processes, are needed to design successful biomaterials for tissue regeneration applications. Currently, attention is focused on the creation of scaffolds with drug-delivery capacity. Scaffolds can represent biofactors' reservoirs, are released during time, and are able to promote cell growth and/or differentiation, allowing more rapid bone healing. On the basis of our results, Sr^2+^ ion released from CMCA hydrogel enhanced bone cell differentiation of the PA2-E12 cell line, accelerating new bone matrix formation. These data suggest that hADSCs, combined with enriched biomaterials able to release *in situ* agents effective in osteogenic differentiation, could represent a successful strategy to develop innovative techniques for bone tissue engineering.

## Figures and Tables

**Table 1:** Release and accumulation of Sr^2+^ in the culture medium from transwells with CMCA enriched with Sr^2+^.

Days of culture	[Sr^2+^] accumulated in the culture medium in presence of CMCA enriched with 3 mM Sr^2+^	[Sr^2+^] accumulated in the culture medium in presence of CMCA enriched with 300 *μ*M Sr^2+^	[Sr^2+^] accumulated in the culture medium in presence of CMCA enriched with 30 *μ*M Sr^2+^	[Sr^2+^] accumulated in the culture medium in presence of CMCA enriched with 3 *μ*M Sr^2+^	Replacement of the old OM with fresh OM
**1**	**1095 ± 33** **mM**	**110 ± 3** ***μ*** **M**	12 ± 0.4 μ**M**	**<3** ***μ*** **M**	
2	1769 ± 68 mM	172 ± 5 *μ*M	19 ± 0.5 *μ*M	<3 *μ*M	
3	2185 ± 76 mM	220 ± 6 *μ*M	20 ± 0.5 *μ*M	<3 *μ*M	X
4	256 ± 8 *μ*M	24 ± 0.7 *μ*M	2.6 ± 0.1 *μ*M	<3 *μ*M	
5	413 ± 8 *μ*M	43 ± 1.1 *μ*M	4.2 ± 0.1 *μ*M	<3 *μ*M	
6	510 ± 14 *μ*M	50 ± 1.6 *μ*M	5.1 ± 0.2 *μ*M	<3 *μ*M	
**7**	**570 ± 16** ***μ*** **M**	**59 ± 1.5** ***μ*** **M**	6.0 ± 0.2 μ**M**	**<3** ***μ*** **M**	**X**
8	37 ± 1 *μ*M	3.6 ± 0.1 *μ*M	<3 *μ*M	<3 *μ*M	
9	59 ± 2 *μ*M	6.0 ± 0.2 *μ*M	<3 *μ*M	<3 *μ*M	
10	73 ± 2 *μ*M	7.2 ± 0.2 *μ*M	<3 *μ*M	<3 *μ*M	X
11	9 ± 0.3 *μ*M	<3 *μ*M	<3 *μ*M	<3 *μ*M	
12	14 ± 0.4 *μ*M	<3 *μ*M	<3 *μ*M	<3 *μ*M	
13	17 ± 0.6 *μ*M	<3 *μ*M	<3 *μ*M	<3 *μ*M	
**14**	**19** ** ± 0.6** ***μ*** **M**	**<3** ***μ*** **M**	**<3** ***μ*** **M**	**<3** ***μ*** **M**	**X**
17	<3 *μ*M	<3 *μ*M	<3 *μ*M	<3 *μ*M	X
21	<3 *μ*M	<3 *μ*M	<3 *μ*M	<3 *μ*M	X
**28**	**<3** ***μ*** **M**	**<3** ***μ*** **M**	**<3** ***μ*** **M**	**<3** ***μ*** **M**	**X**
**42**	**<3** ***μ*** **M**	**<3** ***μ*** **M**	**<3** ***μ*** **M**	**<3** ***μ*** **M**	**X**

Values expressed as means ± SD of Sr^2+^ at times for quantitative ALP and HA analysis are indicated in bold.

## References

[1] Arvidson K, Abdallah BM, Applegate LA (2011). Bone regeneration and stem cells. *Journal of Cellular and Molecular Medicine*.

[2] Ciapetti G, Ambrosio L, Savarino L (2003). Osteoblast growth and function in porous poly *ε*-caprolactone matrices for bone repair: a preliminary study. *Biomaterials*.

[3] Lin ASP, Barrows TH, Cartmell SH, Guldberg RE (2003). Microarchitectural and mechanical characterization of oriented porous polymer scaffolds. *Biomaterials*.

[4] Kneser U, Schaefer DJ, Polykandriotis E, Horch RE (2006). Tissue engineering of bone: the reconstructive surgeon’s point of view. *Journal of Cellular and Molecular Medicine*.

[5] Temenoff JS, Mikos AG (2000). Injectable biodegradable materials for orthopedic tissue engineering. *Biomaterials*.

[6] Deng M, James R, Laurencin CT, Kumbar SG (2012). Nanostructured polymeric scaffolds for orthopaedic regenerative engineering. *IEEE Transactions on Nanobioscience*.

[7] Langer R, Vacanti JP (1993). Tissue engineering. *Science*.

[8] Yang S, Leong KF, Du Z, Chua CK (2001). The design of scaffolds for use in tissue engineering. Part I. Traditional factors. *Tissue Engineering*.

[9] Weinand C, Pomerantseva I, Neville CM (2006). Hydrogel-*β*-TCP scaffolds and stem cells for tissue engineering bone. *Bone*.

[10] Howard D, Buttery LD, Shakesheff KM, Roberts SJ (2008). Tissue engineering: strategies, stem cells and scaffolds. *Journal of Anatomy*.

[11] Weigel T, Schinkel G, Lendlein A (2006). Design and preparation of polymeric scaffolds for tissue engineering. *Expert Review of Medical Devices*.

[12] Caplan AI, Goldberg VM (1999). Principles of tissue engineered regeneration of skeletal tissues. *Clinical Orthopaedics and Related Research*.

[13] Solchaga LA, Goldberg VM, Caplan AI (2001). Cartilage regeneration using principles of tissue engineering. *Clinical Orthopaedics and Related Research*.

[14] Laurencin CT, Ambrosio AMA, Borden MD, Cooper JA (1999). Tissue engineering: orthopedic applications. *Annual Review of Biomedical Engineering*.

[15] Mano JF, Sousa RA, Boesel LF, Neves NM, Reis RL (2004). Bioinert, biodegradable and injectable polymeric matrix composites for hard tissue replacement: state of the art and recent developments. *Composites Science and Technology*.

[16] Suchanek W, Yoshimura M (1998). Processing and properties of hydroxyapatite-based biomaterials for use as hard tissue replacement implants. *Journal of Materials Research*.

[17] Petite H, Vandamme K, Monfoulet L, Logeart-Avramoglou D (2011). Strategies for improving the efficacy of bioengineered bone constructs: a perspective. *Osteoporosis International*.

[18] Langer R (2000). Tissue engineering. *Molecular Therapy*.

[19] Deng M, Nair LS, Nukavarapu SP (2010). In situ porous structures: a unique polymer erosion mechanism in biodegradable dipeptide-based polyphosphazene and polyester blends producing matrices for regenerative engineering. *Advanced Functional Materials*.

[20] Chaikof EL, Matthew H, Kohn J, Mikos AG, Prestwich GD, Yip CM (2002). Biomaterials and scaffolds in reparative medicine. *Annals of the New York Academy of Sciences*.

[21] Friedenstein AJ, Petrakova KV, Kurolesova AI, Frolova GP (1968). Heterotopic of bone marrow. Analysis of precursor cells for osteogenic and hematopoietic tissues. *Transplantation*.

[22] Friedenstein AJ, Piatetzky-Shapiro II, Petrakova KV (1966). Osteogenesis in transplants of bone marrow cells. *Journal of Embryology and Experimental Morphology*.

[23] Pittenger MF, Mackay AM, Beck SC (1999). Multilineage potential of adult human mesenchymal stem cells. *Science*.

[24] Zuk PA, Zhu M, Ashjian P (2002). Human adipose tissue is a source of multipotent stem cells. *Molecular Biology of the Cell*.

[25] Lee OK, Kuo TK, Chen WM, Lee KD, Hsieh SL, Chen TH (2004). Isolation of multipotent mesenchymal stem cells from umbilical cord blood. *Blood*.

[26] De Coppi P, Bartsch G, Siddiqui MM (2007). Isolation of amniotic stem cell lines with potential for therapy. *Nature Biotechnology*.

[27] Gronthos S, Brahim J, Li W (2002). Stem cell properties of human dental pulp stem cells. *Journal of Dental Research*.

[28] Seale P, Asakura A, Rudnicki MA (2001). The potential of muscle stem cells. *Developmental Cell*.

[29] Quarto R, Mastrogiacomo M, Cancedda R (2001). Repair of large bone defects with the use of autologous bone marrow stromal cells. *New England Journal of Medicine*.

[30] Reyes M, Dudek A, Jahagirdar B, Koodie L, Marker PH, Verfaillie CM (2002). Origin of endothelial progenitors in human postnatal bone marrow. *Journal of Clinical Investigation*.

[31] Makino S, Fukuda K, Miyoshi S (1999). Cardiomyocytes can be generated from marrow stromal cells in vitro. *Journal of Clinical Investigation*.

[32] Deng W, Obrocka M, Fischer I, Prockop DJ (2001). In vitro differentiation of human marrow stromal cells into early progenitors of neural cells by conditions that increase intracellular cyclic AMP. *Biochemical and Biophysical Research Communications*.

[33] Schwartz RE, Reyes M, Koodie L (2002). Multipotential adult rogenitor cells from bone marrow differentiation into functional hepatocyte-like cells. *The Journal of Clinical Investigation*.

[34] Connolly JF (1995). Injectable bone marrow preparations to stimulate osteogenic repair. *Clinical Orthopaedics and Related Research*.

[35] Tiedeman JJ, Connolly JF, Strates BS, Lippiello L (1991). Treatment of nonunion by percutaneous injection of bone marrow and demineralized bone matrix: an experimental study in dogs. *Clinical Orthopaedics and Related Research*.

[36] Zhao Y, Lin H, Zhang J (2009). Crosslinked three-dimensional demineralized bone matrix for the adipose-derived stromal cell proliferation and differentiation. *Tissue Engineering A*.

[37] Hong L, Colpan A, Peptan IA, Daw J, George A, Evans CA (2007). 17-*β* estradiol enhances osteogenic and adipogenic differentiation of human adipose-derived stromal cells. *Tissue Engineering*.

[38] Tognarini I, Sorace S, Zonefrati R (2008). In vitro differentiation of human mesenchymal stem cells on Ti6Al4V surfaces. *Biomaterials*.

[39] Drury JL, Mooney DJ (2003). Hydrogels for tissue engineering: scaffold design variables and applications. *Biomaterials*.

[40] Hoffman AS (2001). Hydrogels for biomedical applications. *Annals of the New York Academy of Sciences*.

[41] Lickorish D, Ramshaw JAM, Werkmeister JA, Glattauer V, Howlett CR (2004). Collagen-hydroxyapatite composite prepared by biomimetic process. *Journal of Biomedical Materials Research A*.

[42] Webster TJ, Ergun C, Doremus RH, Siegel RW, Bizios R (2000). Specific proteins mediate enhanced osteoblast adhesion on nanophase ceramics. *Journal of Biomedical Materials Research*.

[43] Webster TJ, Siegel RW, Bizios R (1999). Osteoblast adhesion on nanophase ceramics. *Biomaterials*.

[44] Burdick JA, Anseth KS (2002). Photoencapsulation of osteoblasts in injectable RGD-modified PEG hydrogels for bone tissue engineering. *Biomaterials*.

[45] Williams CG, Kim TK, Taboas A, Malik A, Manson P, Elisseeff J (2003). In vitro chondrogenesis of bone marrow-derived mesenchymal stem cells in a photopolymerizing hydrogel. *Tissue Engineering*.

[46] Carossino AM, Recenti R, Carossino R (2007). Methodological models for in vitro amplification and maintenance of human articular chondrocytes from elderly patients. *Biogerontology*.

[47] Bryant SJ, Anseth KS (2003). Controlling the spatial distribution of ECM components in degradable PEG hydrogels for tissue engineering cartilage. *Journal of Biomedical Materials Research A*.

[48] Leone G, Fini M, Torricelli P, Giardino R, Barbucci R (2008). An amidated carboxymethylcellulose hydrogel for cartilage regeneration. *Journal of Materials Science*.

[49] Ulrich-Vinther M, Maloney MD, Schwarz EM, Rosier R, O’Keefe RJ (2003). Articular cartilage biology. *The Journal of the American Academy of Orthopaedic Surgeons*.

[50] Hamidouche Z, Fromigué O, Ringe J (2009). Priming integrin *α*5 promotes human mesenchymal stromal cell osteoblast differentiation and osteogenesis. *Proceedings of the National Academy of Sciences of the United States of America*.

[51] Kim HW, Knowles JC, Kim HE (2004). Hydroxyapatite/poly(*ε*-caprolactone) composite coatings on hydroxyapatite porous bone scaffold for drug delivery. *Biomaterials*.

[52] Di Silvio L, Bonfield W (1999). Biodegradable drug delivery system for the treatment of bone infection and repair. *Journal of Materials Science*.

[53] Shin H, Jo S, Mikos AG (2003). Biomimetic materials for tissue engineering. *Biomaterials*.

[54] Csaki C, Schneider PRA, Shakibaei M (2008). Mesenchymal stem cells as a potential pool for cartilage tissue engineering. *Annals of Anatomy*.

[55] Garg T, Singh O, Arora S, Murthy RSR (2012). Scaffold: a novel carrier for cell and drug delivery. *Critical Reviews in Therapeutic Drug Carrier Systems*.

[56] Meunier PJ, Roux C, Seeman E (2004). The effects of strontium ranelate on the risk of vertebral fracture in women with postmenopausal osteoporosis. *New England Journal of Medicine*.

[57] Reginster JY, Seeman E, De Vernejoul MC (2005). Strontium ranelate reduces the risk of nonvertebral fractures in postmenopausal women with osteoporosis: Treatment of Peripheral Osteoporosis (TROPOS) study. *Journal of Clinical Endocrinology and Metabolism*.

[58] Marie PJ (2005). Strontium ranelate: a novel mode of action optimizing bone formation and resorption. *Osteoporosis International*.

[59] Marie PJ (2006). Strontium ranelate: a dual mode of action rebalancing bone turnover in favour of bone formation. *Current Opinion in Rheumatology*.

[60] Bonnelye E, Chabadel A, Saltel F, Jurdic P (2008). Dual effect of strontium ranelate: stimulation of osteoblast differentiation and inhibition of osteoclast formation and resorption in vitro. *Bone*.

[61] Marie PJ, Felsenberg D, Brandi ML (2011). How strontium ranelate, via opposite effects on bone resorption and formation, prevents Osteoporosis. *Osteoporosis International*.

[62] Qiu K, Zhao XJ, Wan CX, Zhao CS, Chen YW (2006). Effect of strontium ions on the growth of ROS17/2.8 cells on porous calcium polyphosphate scaffolds. *Biomaterials*.

[63] Song W, Wang Q, Wan C (2011). A novel alkali metals/strontium co-substituted calcium polyphosphate scaffolds in bone tissue engineering. *Journal of Biomedical Materials Research B*.

[64] Liu F, Zhang X, Yu X, Xu Y, Feng T, Ren D (2011). In vitro study in stimulating the secretion of angiogenic growth factors of strontium-doped calcium polyphosphate for bone tissue engineering. *Journal of Materials Science*.

[65] Barbucci R, Leone G, Monici M, Pantalone D, Fini M, Giardino R (2005). The effect of amidic moieties on polysaccharides: evaluation of the physicochemical and biological properties of amidic carboxymethylcellulose (CMCA) in the form of linear polymer and hydrogel. *Journal of Materials Chemistry*.

[66] Beauchemin D (2006). Inductively coupled plasma mass spectrometry. *Analytical Chemistry*.

[67] Hubbell JA (1999). Bioactive biomaterials. *Current Opinion in Biotechnology*.

[68] Healy KE (1999). Molecular engineering of materials for bioreactivity. *Current Opinion in Solid State and Materials Science*.

[69] Sakiyama-Elbert SE, Hubbell JA (2001). Functional biomaterials: design of novel biomaterials. *Annual Review of Materials Science*.

[70] Humphries MJ, Akiyama SK, Komoriya A (1986). Identification of an alternatively spliced site in human plasma fibronectin that mediates cell type-specific adhesion. *Journal of Cell Biology*.

[71] Garg T, Singh O, Arora S, Murthy RSR (2012). Scaffold: a novel carrier for cell and drug delivery. *Critical Reviews in Therapeutic Drug Carrier Systems*.

[72] Rowley JA, Madlambayan G, Mooney DJ (1999). Alginate hydrogels as synthetic extracellular matrix materials. *Biomaterials*.

[73] Lutolf MP, Weber FE, Schmoekel HG (2003). Repair of bone defects using synthetic mimetics of collagenous extracellular matrices. *Nature Biotechnology*.

[74] Verberckmoes SC, Behets GJ, Oste L (2004). Effects of strontium on the physicochemical characteristics of hydroxyapatite. *Calcified Tissue International*.

[75] Lutolf MP, Hubbell JA (2005). Synthetic biomaterials as instructive extracellular microenvironments for morphogenesis in tissue engineering. *Nature Biotechnology*.

